# Carbon Dots in Nanomedicine: Advanced Fabrication, Biomedical Applications, and Future Clinical Perspectives

**DOI:** 10.3390/pharmaceutics18050632

**Published:** 2026-05-21

**Authors:** Muhammad Sohail Khan, Imran Zafar, Dayeon Ham, Ki Sung Kang, Il-Ho Park

**Affiliations:** 1 College of Korean Medicine, Gachon University, 1342 Seongnamdaero, Seongnam 13120, Republic of Korea; sohail@gachon.ac.kr; 2 Department of Biochemistry and Biotechnology, Faculty of Science, The University of Faisalabad (TUF), Faisalabad 38000, Pakistan; bioinfo.pk@gmail.com; 3 College of Literature, Science, and the Arts, University of Michigan, Ann Arbor, MI 48109, USA; liah@umich.edu; 4 College of Pharmacy, Sahmyook University, Seoul 01795, Republic of Korea

**Keywords:** carbon dots, CQDs, nanomedicine, targeted drug delivery, bioimaging, clinical translation

## Abstract

Carbon dots (CDs), including carbon quantum dots (CQDs), are ultra-small carbon-based nanomaterials, typically below 10 nm, with tunable photoluminescence, high aqueous dispersibility, favorable biocompatibility, low toxicity, and abundant surface functional groups. These properties make CDs promising multifunctional platforms for nanomedicine, particularly in bioimaging, biosensing, targeted drug/gene delivery, photodynamic therapy (PDT), photothermal therapy (PTT), antimicrobial treatment, and theranostic applications. This review critically examines recent advances in CD fabrication, including top-down, bottom-up, green biomass-derived, microwave-assisted, hydrothermal, and emerging hybrid strategies, with emphasis on how precursor selection, heteroatom doping, surface passivation, and polymer/ligand functionalization regulate optical performance, biological interaction, and therapeutic efficiency. The review discusses structural classification, including CQDs, graphene quantum dots (GQDs), carbon nanodots, and carbonized polymer dots (CPDs), together with major characterization approaches such as ultraviolet–visible (UV–Vis) spectroscopy, Fourier-transform infrared (FTIR) spectroscopy, X-ray diffraction (XRD), X-ray photoelectron spectroscopy (XPS), Raman spectroscopy, and high-resolution transmission electron microscopy (HRTEM). Particular attention is given to red/near-infrared (NIR) emission, renal clearance, drug-loading behavior, reactive oxygen species (ROS) generation, toxicity mechanisms, biodistribution, and long-term biosafety. This review also highlights key translational barriers, including batch-to-batch variability, limited standardization, scalable manufacturing, regulatory uncertainty, and incomplete pharmacokinetic evaluation. It considers artificial intelligence (AI) and machine learning (ML) as emerging tools for reproducible CD design. CDs represent versatile and clinically promising nanoplatforms, but their translation requires standardized synthesis, rigorous safety assessment, and application-specific regulatory validation.

## 1. Introduction

Nanotechnology in medicine (or nanomedicine) uses nanoparticles and nanoscale materials to enhance diagnostics, drug delivery, medical imaging, and therapeutic interventions [1,2,3]. In cancer and infectious disease treatment, nanoparticles allow for targeted drug delivery, optimizing the efficiency of treatments and reducing adverse effects [4,5]. The use of contrast-enhancing nanoparticles based on new optical and magnetic properties has greatly improved medical imaging [6,7]. Its potential in personalized medicine, regenerative medicine, and clinical diagnostics is promising, but there are concerns about nanotoxicity, environmental effects, and long-term safety that need careful attention before it can be broadly translated to the clinic [2,8,9,10].

Carbon dots (CDs) are carbon-based nanomaterials that are generally less than 10 nm in size, and are prepared by both top-down and bottom-up approaches, for example, by arc discharge and laser ablation, as well as by hydrothermal and microwave-assisted synthesis, which allow for precise control of particle size and optical properties [11]. CDs possess a high-water solubility, tunable photoluminescence, good biocompatibility, low toxicity, and abundant surface functional groups, including amino, hydroxyl, and carboxyl moieties, which enable functionalization of the surfaces for various biomedical applications [12]. Their unique optical and electronic characteristics, including high quantum yield, photostability, and efficient electron transfer, have made them promising candidates for bioimaging, biosensing, drug delivery, phototherapy, and energy-related applications [13,14].

Metal–organic framework (MOF)-derived carbons are highly attractive due to their large specific surface area, adjustable porosity, adjustable composition, and outstanding adsorption and catalytic activities [15]. High-temperature carbonization of MOFs is a common method for producing these porous carbon materials, which have various applications, such as energy storage, electrocatalysis, and environmental remediation [16,17]. For instance, nitrogen-doped porous carbons prepared by loading melamine into MAF-6 were shown to exhibit excellent adsorption properties for nitroimidazole antibiotics via electrostatic interactions, hydrogen bonding, and π–π stacking [18]. Notwithstanding these benefits, MOF-derived carbons differ in structure from CDs.

MOF-derived carbons are typically bulk materials that are mesoporous or microporous and have been optimized for adsorption and catalysis. At the same time, CDs are ultrasmall carbon nanoparticles (2–10 nm) with a high density of functional groups (–COOH, –OH, –NH_2_) that make them highly water-dispersible and readily biofunctionalizable with minimal cytotoxicity [19,20]. Moreover, CDs, unlike larger porous carbons derived from MOFs, can be cleared by the kidneys efficiently for biomedical and intravenous applications if the hydrodynamic diameter of the particles is below the glomerular filtration threshold [21]. Thus, MOF-derived carbons and CDs can be considered as complementary carbon nanomaterials; while MOF-derived carbons are better for catalysis and adsorption, CDs are better for fluorescence imaging, biosensing, drug delivery, and theranostic applications.

Compared with other biomedical nanomaterials, CDs have several important advantages, including intrinsic fluorescence, excellent dispersibility in aqueous media, low heavy-metal toxicity, facile surface modification, and high photostability [22,23]. In addition, CDs exhibit tunable photoluminescence and high photothermal conversion efficiency, making them suitable for bioimaging, photodynamic therapy, and photothermal therapy [24]. Metal oxide nanoparticles, on the other hand, can release toxic metal ions under physiological conditions, and polymeric nanomaterials may have to be functionalized with extra fluorescent dyes due to their lack of inherent optical properties.

Graphene oxide and carbon nanotubes exhibit high drug loading and photothermal efficiency, but can also be linked to slow clearance, oxidative stress, and inflammatory toxicity due to their large size and high aspect ratio [25]. However, CDs also have several drawbacks, including batch-to-batch variability, limited understanding of fluorescence mechanisms, and lower quantum yields in red/NIR fluorescent systems [26]. Biodistribution, long-term toxicity, and regulatory standardization are other challenges for clinical translation. Therefore, CDs should not be regarded as the best type of nanomaterial for all applications, but rather as a material that has specific benefits in terms of fluorescence tracking, low toxicity, renal clearance, and multifunctional theranostic performance in a single nanoplatform [27].

Recent studies have examined CDs and carbon quantum dots (CQDs) across several isolated domains. Bartkowski et al. [28] focused on CD synthesis, bioimaging, and anticancer drug delivery; Shen et al. [29] reviewed targeted bioimaging and cancer theranostics; Pechnikova et al. [19] addressed advances and challenges in CQD biomedical applications, including drug delivery, biosensing, and phototherapy; and [30] explored AI-assisted green CD synthesis and intelligent drug delivery. Despite these contributions, each review addresses a single application domain or enabling strategy without connecting fabrication route, structural subtype, heteroatom/surface engineering, photophysical mechanism, biomedical function, safety profile, scalability, and regulatory readiness within a unified translational framework. Bartkowski et al. [31] highlighted the urgent need for standardized synthesis and characterization to overcome regulatory and clinical translation barriers. This integrated, mechanism-to-translation perspective constitutes the defining gap addressed by the present review.

The field of carbon dot (CD) research is rapidly progressing from proof-of-concept synthesis toward the rational design of clinically relevant, application-oriented nanomaterials. Although previous reviews have addressed CD synthesis, biomedical applications, cancer theranostics, toxicity, and artificial intelligence (AI)-assisted optimization, a comprehensive mechanism-to-translation perspective remains insufficiently developed. This review, therefore, examines the complete developmental pathway of CDs, including precursor selection, synthesis strategy, structural classification, crystalline versus amorphous architecture, surface modification, heteroatom doping, photophysical behavior, biomedical function, biosafety, manufacturing scalability, and clinical translation potential. Its central contribution lies in integrating structure–property–function relationships with translational requirements, thereby clarifying how synthesis-controlled structural features influence optical performance, therapeutic efficacy, toxicity profile, and clinical feasibility. The review also discusses emerging AI-driven design and optimization strategies as tools for improving reproducibility, safety, and application-specific performance in future CD-based nanomedicine platforms.

## 2. Classification and Physicochemical Properties of Carbon Dots

### 2.1. Physicochemical Properties

**Figure 1:**
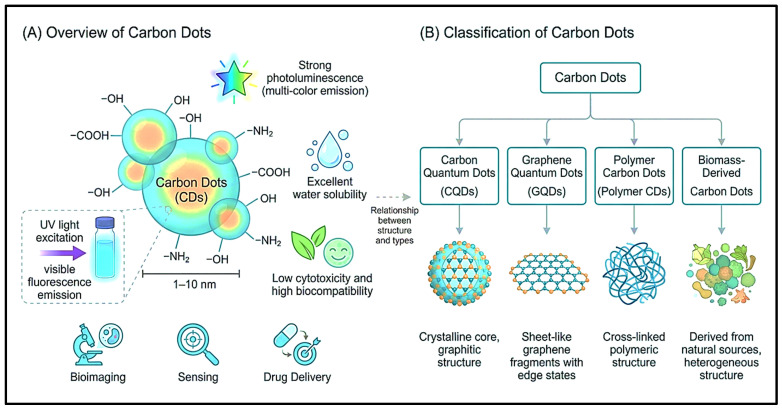
Overview of Structural Diversity and Classification of CDs. (**A**) Key Physicochemical Properties of CDs. (**B**) Structural Classification of CDs Subtypes.

Carbon dots (CDs) constitute a class of zero-dimensional (0D) carbon-based nanomaterials typically confined to a size range of 1–10 nm, characterized by a dual sp^2^/sp^3^-hybridized carbon framework whose optical, electronic, and biological behaviour is fundamentally governed by the interplay between core crystallinity, surface chemistry, and heteroatom doping state, as seen in as shown in Figure 1A. These materials are highly attractive for diagnostics, therapeutics, and nanomedicine owing to their remarkable optical and physicochemical properties, including photoluminescence, biocompatibility, and aqueous dispersibility [32]. CDs possess favourable attributes, including abundant functional groups (e.g., amino, hydroxyl, and carboxyl), high colloidal stability, and significant electron mobility, all of which collectively underpin their broad utility across optical and biomedical domains [33]. Their unique physicochemical properties make them well-suited for bioimaging, drug delivery, biosensing, and photodynamic therapy [30]. The optical properties of CDs are highly photostable and exhibit tunable fluorescence that can be fine-tuned by adjusting particle size, surface states, and heteroatom doping, thereby modulating their electronic structure and photoluminescent response [34].

### 2.2. Structural Architecture and Core Crystallinity

The internal structural organization of CDs spans a spectrum from amorphous CPDs to partially graphitized and fully graphitic carbon quantum dots (GQDs), with crystallinity dictating their photophysical response as detailed in Figure 1B. X-ray diffraction (XRD) patterns of graphitized CDs exhibit a characteristic peak centered at 2θ ≈ 27°, corresponding to the (002) graphitic plane with a d-spacing of approximately 0.35 nm, reflecting sp^2^-hybridized carbon arranged in a graphitic lattice; in contrast, amorphous CDs display only broad background intensity arising from highly disordered carbonaceous material [35]. A broad diffraction peak centered near 2θ ≈ 25° is universally observed in carbon-based nanomaterials and serves as a diagnostic indicator of amorphous or low-crystalline graphitic-like carbon [36]. XRD diffractograms of CQDs also show broad peaks centered at 2θ ≈ 21°, with an interlayer spacing of approximately 0.34 nm, indicative of structural disorder arising from surface oxygen-containing functionalities [37]. After prolonged hydrothermal carbonization, the XRD spectrum of nitrogen-doped CDs (NCDs) commonly illustrates a broad peak at approximately 21.1°, indicative of an amorphous turbostratic carbon phase, with HRTEM-derived d-spacings near 0.26 nm corresponding to the (100) graphitic carbon plane [37]. Thermogravimetric and X-ray photoelectron spectroscopy (XPS) analyses confirm that higher pyrolysis temperatures promote progressive carbonization, with the O/C ratio decreasing systematically and C 1s deconvolution showing an increasing sp^2^ contribution indicative of evolving turbostratic graphitic domains [38].

The Raman spectroscopic signature of CDs universally presents two principal vibrational modes: the D band (~1350 cm^−1^), reflecting sp^2^ microdomains with bond-angle disorder induced by sp^3^ hybridization, and the G band (~1580 cm^−1^), attributed directly to in-plane stretching of sp^2^ C–C bonds [39]. The I_D_/I^G^ intensity ratio thus serves as a quantitative metric of structural defect density, with higher ratios reflecting greater structural disorder. HRTEM corroborates core structure at the nanoscale, revealing lattice fringes in crystalline CDs and confirming amorphous morphology in carbonized polymer-type variants. At the same time, selected area electron diffraction (SAED) patterns distinguish crystalline from amorphous nanoarchitectures [40]. Critically, all distinctive optical properties of CDs, including the giant Stokes shift and excitation-wavelength-dependent emission, arise from the linear optical response of partially sp^2^-hybridized surface domains on an sp^3^-hybridized amorphous core, with the domain hybridization factor governing electron localization and the electronic bandgap within each emissive domain [41].

### 2.3. Surface Chemistry and Functional Group Analysis

Their surface chemistry governs CDs’ aqueous behavior, bioconjugation capacity, and cellular interaction profile. FTIR spectroscopy routinely confirms O–H stretching (~3200–3400 cm^−1^), C=O carbonyl stretching (~1700–1730 cm^−1^), C–O–C ether linkages (~1050–1200 cm^−1^), N–H bending (~1550–1650 cm^−1^), and C–N stretching (~1300–1400 cm^−1^). In particular, the band located at about 1200 cm^−1^ indicates C–O stretching vibrations typical of alcohols, ethers, or esters, and the band at 1300–1400 cm^−1^ points towards the presence of C–N stretching vibrations, which are typical of amine or amide functionalities [36]. The high solubility of CDs in polar solvents arises from the presence of abundant oxygen-containing surface groups such as epoxide, hydroxyl, carbonyl, carboxyl, etc., which not only introduce various energy levels of surface defect states but also provide a variety of transition pathways for electrons, leading to a variety of PL properties [42]. The carboxylic acid groups enable easy functionalization and water dispersion, which is necessary for biomedical applications [37].

XPS provides quantitative elemental composition and bonding state analysis, with C 1s peak deconvolution resolving sp^2^ (C–C/C=C, ~284.5 eV), sp^3^ (C–C, ~285.2 eV), C–O (~286.5 eV), C=O (~287.5 eV), and O–C=O (~288.5–289.0 eV) contributions. Nitrogen doping creates pyridinic-N (~398.5 eV), pyrrolic-N (~400.0 eV), and graphitic-N (~401.3 eV) features that each impart unique changes to the local electronic density of states and the photoluminescence characteristics [40]. HRTEM, XRD, FTIR, Raman, and XPS studies of this multi-technique synthesized N, S-co-doped CDs Venkatesh et al. [40] confirm the formation of monodispersed spherical particles of ~4.8 nm using a one-pot microwave-assisted route, which exhibit an amorphous carbon phase and heteroatom-enriched surface states. The ratio of sp^2^ to sp^3^ hybridization is a common structural parameter that can be determined by deconvolving C 1s XPS spectra and measuring Raman I_D_/I^G^ for carbon nanomaterials, and influences the material’s optical, electronic, and catalytic properties [39,43].

### 2.4. Photoluminescence Mechanisms and Quantum Yield

The mechanism of PL in CDs is complex and is still under study. Three basic models have been proposed: (1) a bandgap transition model based on the quantum confinement of conjugated π-domains within cores containing graphene fragments, which results in the creation of size-dependent emission; (2) a surface state emission model, where the oxidation, doping, or structural defects at the surfaces trap radiative recombination centers for excited electron–hole pairs; and (3) molecular fluorophore models—assuming that the emission is generated by discrete chromophoric units embedded in or anchored to the carbon matrix [44]. The CDs are hydrothermally synthesized and chromatographically separated with tunable PL with emission peaks ranging between 440 and 625 nm, which was attributed to the progressive decrease in the bandgap energy, due to a gradual increase in the oxygen species in the surface architecture, indicating the critical role of the surface oxidation state in tuning the emission color of the CDs [45]. On the other hand, the emission of blue light from the CDs has been attributed to surface states arising from C–O and C=O groups in glucose, and the green light emission to deeper energy levels arising from O–C=O groups [46]. The underlying photophysical mechanism of fluorescence modulation involves quantum confinement, surface-state functionalization, and heteroatom doping [47]. Heteroatom doping is particularly effective for broadening the photoluminescent response: co-doping with two heteroatoms, nitrogen and boron, or nitrogen and phosphorus, yields CDs with significantly enhanced biocompatibility and higher photoluminescence quantum yields compared to single-dopant variants [42]. Surface state engineering has uncovered four distinct PL mechanisms that enable systematic property optimization (SYMPO) studies: quantum confinement effects, surface state hybridization, molecular-state fluorescence, and crosslink-enhanced emission [48]. Additional modulation of the fluorescence color comes from electronic bandgap transitions of π-domains, local surface defect states, discrete fluorophores, and from element-specific doping effects [49]. The ratio of radiative to non-radiative recombination, which is directly influenced by the extent of surface oxidation, the conjugation of the carbon core, and the type of functional groups that protect the surface of the carbon, determines the photoluminescence quantum yield (PLQY) in CDs [44].

### 2.5. Renal Clearance, Colloidal Stability, and Drug Loading

The sub-10 nm size range of the CDs provides a pharmacokinetic benefit, whereby these particles can be excreted from the systemic circulation via glomerular filtration in the kidney, an ideal property for removing potentially toxic nanomaterials [50]. Fluorescence quantum yields of around 58% and high drug loading capacity without toxicity to normal renal tubular cells were shown by renal-clearable sucrose-derived CDs, which were also shown to accumulate at the tumor site for a longer duration in vivo, with doxorubicin-loaded CDs having greater anticancer activity and lesser systemic toxicity in renal cell carcinoma models than free doxorubicin [50]. Administration via the intravenous (IV) route may result in the accumulation of CQDs in organs such as the liver and kidneys. However, many CDs are designed to be excreted in the kidneys without accumulating in organs, thus limiting the potential for chronic toxicity from organ accumulation [19].

The engineering methods that can be employed include the use of non-toxic precursors, enhancing biodegradability, and advancing targeting to reduce off-target effects [19]. Ionized surface carboxyl (pKa ≈ 4.5–5.5) and amino groups provide electrostatic repulsion to keep the particles from aggregating under physiological conditions, resulting in a zeta potential of typically −20 to −40 mV at near neutral pH. The physicochemical properties of CDs are stable, and their surface chemistry can be controlled and modified, while they also possess high biocompatibility and strong photoluminescence, making them useful multifunctional biomedical platforms in conditions close to those of living cells (pH, ionic strength, salt) [51]. Finally, engineered surface-state CD is designed to produce a consistent emission response across excitation wavelengths from 280 nm to 480 nm, enabling stable performance in complex biological settings for bioimaging. In addition, drug delivery capacity is achieved through functionalization with targeting molecules, such as surface functionalization with targeting moieties for large neutral amino acid transporter 1 (LAT1), which allows selective tumor accumulation in xenograft models [52]. The size of the quantum-sized CDs is tunable; they can be surface-functionalized to achieve targeted delivery of a therapeutic agent, and they are biocompatible, thereby strengthening anticancer activity, as demonstrated by simultaneous fluorescence imaging in cancer cells [53].

### 2.6. Structural Classification and Characterization Summary

CDs are broadly subclassified into graphene quantum dots (GQDs), CQDs with defined crystal lattice structures, and carbon nanodots or carbonized polymer dots (CNDs/CPDs) that are largely amorphous—each subclass exhibiting distinct XRD fingerprints, Raman I_D_/I^G^ ratios, HRTEM lattice fringe patterns, and XPS elemental bonding profiles that collectively dictate their quantum yield, emission wavelength tunability, drug loading efficiency, and in vivo biocompatibility profile. The photoluminescence of CDs depends on a convergence of synthesis method, precursor composition, surface states, and heteroatom doping; oxygen-containing functionalities particularly carboxylic acid moieties provide a substrate for facile surface functionalization and stable aqueous dispersion [37]. Advanced structural characterization confirms that CQDs possess strong fluorescence, particularly in the red and near-infrared (NIR) spectral regions, rendering them well-suited for deep-tissue bioimaging. At the same time, their versatile surface chemistry supports targeted drug delivery, antimicrobial applications, and integrated theranostic platforms [54]. Variability in synthesis protocols, inconsistent batch-to-batch reproducibility, and insufficient data on long-term toxicity and biodistribution remain significant challenges impeding clinical translation [19]. These collective structural and surface features directly influence fluorescence quantum yield, emission wavelength, photostability, drug-loading efficiency, protein corona formation, and in vivo toxicity profiles, parameters that must be optimized holistically for successful clinical translation of CD-based nanomedicines.

## 3. Carbon Dot Fabrication: Top-Down, Bottom-Up, and Emerging Hybrid Strategies

**Figure 2:**
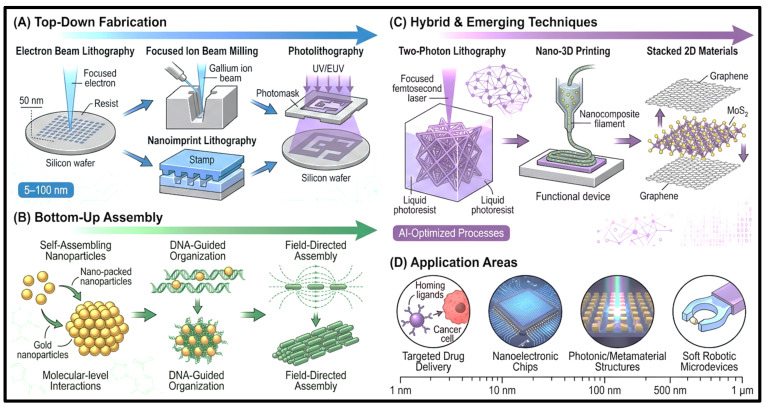
Advanced Nanofabrication Strategies: Integration of Top-Down, Bottom-Up, and Emerging Hybrid Approaches for Functional Nanosystems. (**A**) Top-down nanofabrication techniques for high-precision patterning of nanoscale structures; (**B**) bottom-up assembly strategies for controlled organization of nanomaterials into hierarchical architectures; (**C**) emerging hybrid nanofabrication techniques, including two-photon lithography and nanoscale additive manufacturing; and (**D**) AI-assisted and integrated nanomanufacturing for enhanced precision, scalability, and functional device fabrication.

CD fabrication is commonly achieved through top-down and bottom-up strategies using ‘top down’ and ‘bottom up’ approaches to build nanoscale structures with tunable physicochemical properties, as shown in Figure 2. The top-down methods include oxidation, electro-oxidation, and physical disintegration to separate bulk carbonaceous materials into nanosized CD fragments, as shown in Figure 2A. Common techniques include arc discharge, laser ablation, electrochemical exfoliation of graphite, carbon nanotubes, graphene oxide, and fullerenes. Acid oxidation (HNO_3_, H_2_SO_4_, mixture of these) results in the introduction of oxygen-containing functional groups (carboxyl (−COOH), hydroxyl (−OH), epoxy (−C−O−C−), which increases the colloidal stability and allows for additional bioconjugation. By tuning the fluence, pulse duration, and solvent environment, fine control of particle size (typically 2–5 nm) and oxidation level (typical surface oxidation) can be achieved using laser ablation. In contrast, an electrochemical exfoliation can be used to produce fine particles on a large scale with tunable surface oxidation and quantum yield via applied potential.

Nevertheless, top-down approaches are generally plagued by large size distributions, batch-to-batch variations, and poor control of heteroatom doping. Bottom-up processes involve hydrothermal, solvothermal, microwave-assisted, and pyrolytic carbonization with molecular assembly as seen in Figure 2B. The most commonly used method is hydrothermal synthesis (120–250 °C), which can be performed with a variety of precursors, including citric acid, urea, glucose, ethylenediamine, and amino acids, because they are readily available, inexpensive, and have excellent doping ability. The incorporation of N, S, P, or B in situ results in a significant modification of the electronic structure that leads to a significant increase in the photoluminescence of the CDs, with quantum yields of CDs co-doped with N and S (CDs + N + S→ N, S − CDs) reaching QY ≈ 40–80% under optimized conditions [55].

Microwave-assisted synthesis also promotes faster reactions through rapid dielectric heating, and solvothermal reactions in high-boiling solvents (e.g., Dimethylformamide (DMF), Ethylene glycol (EG) = HO–CH_2_–CH_2_–OH) lead to greater π-conjugation and a red-shifted emission [56]. The pyrolysis-based route is simple, but sometimes post-synthetic purification is needed because of the wide size distribution. Conventional fabrication methods remain limited by reproducibility, scalability, and surface-control challenges, prompting the development of alternative methods, such as hybrid fabrication and AI-based fabrication, as shown in Figure 2C,D. Microfluidic systems can perform continuous-flow synthesis, providing better control over reaction time, temperature, and precursor mixing, thereby enhancing the reproducibility and scalability of the process. In addition, template-assisted methods using mesoporous silica, metal–organic frameworks, and cyclodextrins can control the growth of CDs, enabling the production of uniform particle morphology and controlled architectures.

Meanwhile, machine learning methods, including random forest, gradient boosting, and neural networks, have been employed to correlate synthesis parameters (precursor type, pH, temperature, time, and solvent) with optical outputs, such as emission wavelength, quantum yield, and particle size, allowing data-driven optimization of the synthesis, in addition to traditional trial-and-error approaches. Together with standardized synthesis and high-throughput characterization, these are paving the way toward reproducible CD engineering for application-specific design. Bottom-up hydrothermal and microwave-assisted methods are most popular in biomedical research because they work in aqueous media, operate at relatively low temperatures, and can incorporate the carbonization, passivation, and heteroatom doping in a single step using biocompatible precursors [57]. Top-down approaches are less popular in biomedical applications, in which the harsh environment and the less precise control of surface chemistry and dopant incorporation make bottom-up approaches more attractive for achieving biocompatibility, functionalization, and stable optical performance [58].

### 3.1. Surface Functionalization Strategies of Carbon Dots for Biomedical Applications

The surface functionalization of CDs can be carried out using covalent, non-covalent, polymer-assisted, ligand-mediated, heteroatom-doping, and hybrid composite approaches, thereby further enhancing their physicochemical properties and biomedical relevance. These strategies contribute to the improved bioimaging, biosensing, drug delivery, and theranostic capabilities of CDs, underpinning their broader applications in these domains [19,59].

**Figure 3:**
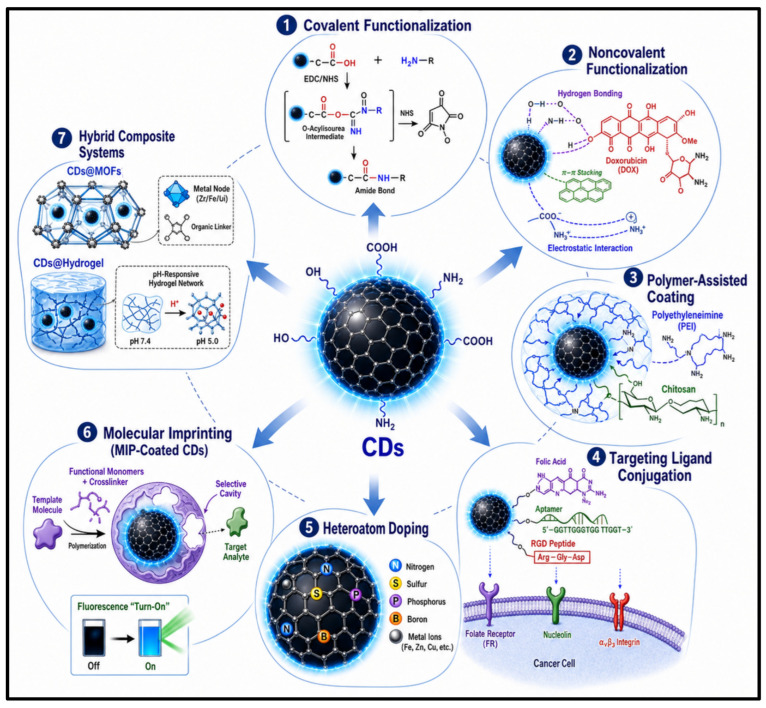
Major Surface Functionalization Strategies of CDs for Enhanced Biomedical Applications.

Covalent functionalization is considered one of the most stable and reliable of all surface engineering techniques. Most of the time, the surface of CDs contains amino, hydroxyl, and carboxyl groups, which can be reacted with amide, ester, sulfonylation, silylation, and copolymerization reactions. Of these, amide coupling (via 1-ethyl-3-(3-dimethylaminopropyl) carbodiimide/N-hydroxysuccinimide (EDC/NHS) is the most commonly used method for bioconjugation. For example, hyaluronic acid was coupled to amine-functionalized CDs using EDC/NHS chemical coupling, with the carboxyl groups activated by EDC and NHS, thereby ensuring higher coupling efficiency and preventing undesirable side reactions. These covalent modifications can enhance the colloidal stability and compatibility in biological systems [59,60]. A versatile and reversible approach, called noncovalent functionalization, is introduced that preserves the intrinsic fluorescence of CDs. Surface interactions, including hydrogen bonding, electrostatic interactions, and π–π stacking, can be used to attach biomolecules and drugs to the carbon core without interfering with it. For drug-loading applications (doxorubicin), the most important criterion is the preservation of the fluorescence, for simultaneous imaging and therapy [19]. A polymer-based functionalization method has proven highly effective in improving the performance of CDs. The surface of the CDs was modified with polyethyleneimine (PEI) to achieve high photoluminescence quantum yields, excellent photostability, and increased cellular uptake, owing to the presence of positive charges. The studies have also shown that the viability of fibroblasts is well maintained, with viability above 80% up to 1000 µg/mL of PEI-derived CDs. All the same advantages are obtained for the chitosan coatings, with respect to stability, biocompatibility, and drug encapsulation efficiency. The polymeric coating with glutaraldehyde crosslinking was found to achieve a drug encapsulation efficiency of over 96% for boron-silane-doped CDs, highlighting the critical role of polymeric coatings in biomedical delivery systems [19]. The strategy of targeting ligand conjugation makes the CDs an active theranostic nanoplatform that specifically targets cells. The folate-positive CDs specifically recognize the folate receptor (FR) that is over-expressed in cancerous cells; thus, receptor-mediated endocytosis is triggered. Likewise, it has been demonstrated that AS1411-conjugated gadolinium-doped CDs can significantly enhance magnetic resonance imaging (MRI) contrast in adenocarcinoma cells by targeting nucleolin. RGD peptide-functionalized CDs are also shown to mediate integrin-dependent uptake in tumor tissue. The targeting specificities of these ligands and the therapeutic effect of cancer imaging and therapy are significantly enhanced [61]. One of the most important methods for modifying the intrinsic electronic and optical properties of CDs is heteroatom doping. Doping with elements such as N, S, P, B, F, and transition metals (TM) alters the electron distribution and surface defect states, thereby improving fluorescence intensity, quantum yield, and photostability. Co-doping with N and metal ions such as Mn, Zn, Cu, Gd, and Ni has been shown to induce a synergistic effect on optical and catalytic properties. For instance, the absolute fluorescence quantum yield of Ni-doped CDs has been increased to 54.7% by increasing graphitization and reducing nonradiative recombination pathways [62,63]. A novel recognition method for CD functionalization has been introduced: Molecular imprinting. MIP-coated CDs could provide a new platform for the fluorescence-based sensing of target analytes, such as glucose and sialic acid, with greater selectivity. CDs in silica or MIP matrices exhibit fluorescence turn-on sensing properties and have been shown to recognize cancer cells with improved specificity compared to passive targeting systems [64]. The newest development in the field of CD surface engineering is the hybrid composite systems. The carbon dot–metal–organic framework (CDs@MOFs) hybrid possesses the inherent porosity and tunable architecture of MOFs, in addition to the fluorescence and biocompatibility of CDs, making it an attractive material for multifunctional biomedical applications. Similarly, pH-dependent luminescent properties are also presented in the CD–hydrogel composites, and they can be applied to monitoring sweat, optical communication, and drug delivery. In addition to the multifunctional hydrogels, the nanozyme activity and UV-shielding properties also highlight the versatility of the hybridized CD systems [65]. To broaden their application potential in advanced biomedical fields, the optical, chemical, and biological properties of CDs are improved through key surface functionalization methods, including covalent modification, noncovalent interactions, polymer-assisted coating, ligand conjugation, heteroatom doping, molecular imprinting, and hybrid composite formation, as illustrated in Figure 3.

### 3.2. Precursor-to-Property Engineering of Carbon Dots for Biomedical Translation

The sequence of steps involved in the bottom-up formation of CDs can be summarized as hydrolysis/dehydration of the precursor or precursors, condensation/polymerization, nucleation, carbonization, and surface passivation. These changes are most important in hydrothermal systems where high temperature and autogenous pressure cause the carbon core to develop gradually and the surface states to develop for optical behavior. All reaction conditions, including the temperature, reaction time, polarity of the solvents, the ratio of precursors, and reaction pH, determine the extent of carbonization, the size of the sp^2^-domains, the surface defect density, the incorporation of the dopant, and, consequently, the fluorescence quantum yield (QY), emission wavelength, excitation dependence, and biological performance. Heteroatom doping has a strong influence on the electronic and emissive properties. Nitrogen-containing precursors such as urea and ethylenediamine can give rise to pyridinic, pyrrolic, graphitic, and amine-like surface states. At the same time, sulfur sources such as thiourea and cysteine can introduce thiol, sulfonate, and C–S/C=S functionalities, which alter charge distribution and radiative recombination pathways.

Recently, N, S co-doped CDs based on citric acid and cysteine have been reported, with quantum yields up to 73%, which are attributed to synergistic doping effects that inhibit non-radiative decay and generate additional emissive centers. [66]. Nitrogen precursors also influence the optical output, as urea- and tris-based systems often yield more uniform CDs and are less dependent on excitation wavelength than amino acids such as β-alanine or chelating agents such as EDTA, which may lead to broader size distributions and excitation-dependent emission profiles [67]. However, as outlined in Table 1, the synthesis parameters have a direct influence on the structure–property relationships of the CD, which means that fabrication is a key step in determining the core architecture, surface chemistry, emissive states, and ultimately the biological performance of the CD and is not just a preparative step.

## 4. Advanced Characterization Techniques for Carbon Dots

**Figure 4:**
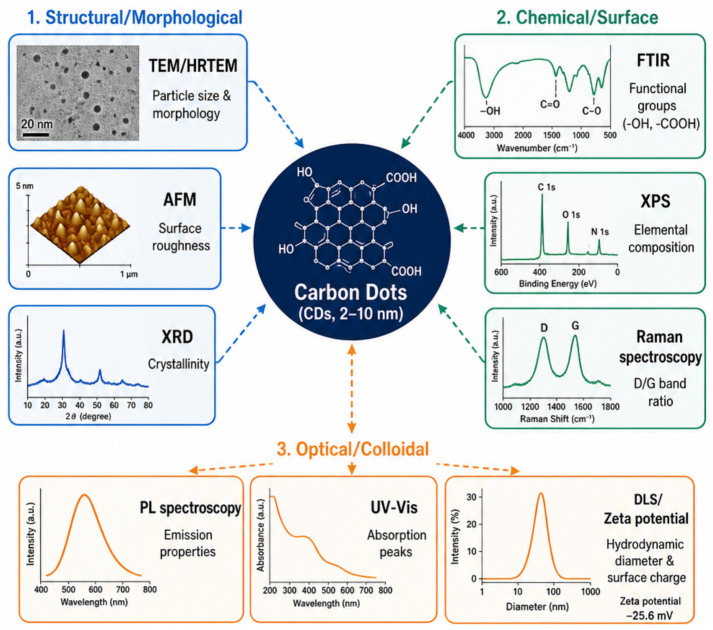
Structural, Optical, and Surface Characterization Techniques for CDs.

Carbon dots (CDs), typically 2–10 nm in size, possess remarkable optical properties, high biocompatibility, and excellent cellular penetrability, making them promising materials for biomedical imaging, sensing, drug delivery, optoelectronics, and photocatalysis, as illustrated in Figure 4. Comprehensive characterization of CDs relies on a combination of spectroscopic, microscopic, and analytical techniques to assess their structural, chemical, and optical properties. Among the most common methods, UV-Vis spectroscopy and photoluminescence spectroscopy can be used to investigate absorption and emission properties and to recover information on electronic transitions and fluorescence dynamics [77,78,79]. Fourier-transform infrared (FTIR) and Raman spectroscopy techniques provide information on surface functional groups and structural defects, which affect photoluminescence and chemical reactivity [80,81]. The determination of particle size, morphology, and crystallinity is performed with the help of an HRTEM, and the elemental composition and bonding states on the CD surface are identified with the help of XPS [77,82].

The detailed molecular structures can be elucidated using nuclear magnetic resonance (NMR) spectroscopy, supported by density functional theory (DFT) modeling, and by differentiating between polymer-like and carbon-core components in CDs [83]. Furthermore, purification and fractionation are performed using methods such as dialysis, chromatography, and centrifugation to obtain homogeneous samples of CDs for accurate characterization [84,85]. Advanced single-particle characterization techniques have also emerged to resolve nanoscale heterogeneity, dopant distribution, and non-crystalline structural variations that are often obscured in ensemble-average analyses. A comparative summary of CDs derived from natural and synthetic precursors, including their characterization techniques, key physicochemical properties, and biomedical applications, is presented in Table 2.

## 5. Biomedical Applications of Carbon Dots: Diagnostic and Therapeutic Platforms

Carbon dots (CDs), including CQDs, have emerged as multifunctional nanomaterials for biomedical diagnostics, therapeutic applications, and integrated theranostic systems due to their tunable photoluminescence, ultrasmall particle size, excellent aqueous dispersibility, abundant surface functional groups, and comparatively favorable biocompatibility. These physicochemical characteristics enable their extensive utilization in fluorescence bioimaging, biosensing, targeted drug delivery, antimicrobial treatment, tissue engineering, and regenerative medicine. In fluorescence bioimaging, CDs exhibit strong, stable emission properties, enabling real-time visualization of cellular structures and intracellular processes with minimal photobleaching and cytotoxicity. In biosensing applications, CDs serve as highly sensitive fluorescent nanoprobes for detecting biomolecules, nucleic acids, proteins, glucose, metal ions, and disease biomarkers via mechanisms such as fluorescence quenching, electron transfer, and Förster resonance energy transfer (FRET). Furthermore, surface-functionalized CDs act as efficient nanocarriers for targeted and stimuli-responsive drug delivery. CDs also exhibit antibacterial and antiviral activities through ROS generation and membrane disruption, while their favorable surface properties support tissue engineering, cell proliferation, and regenerative medicine applications.

**Figure 5:**
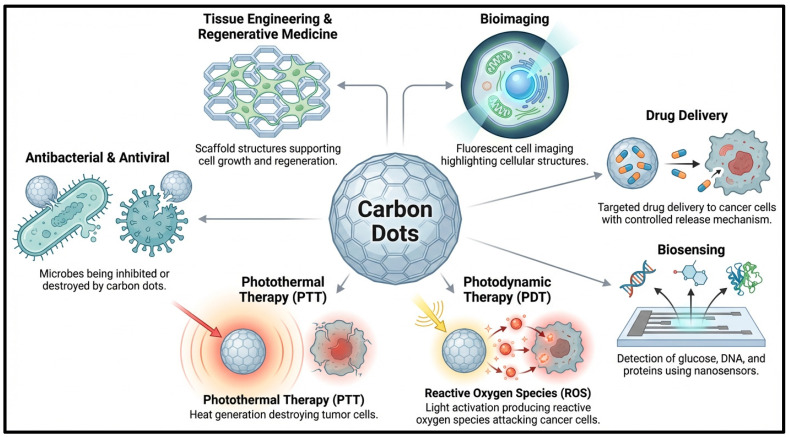
Multifunctional Biomedical Applications of CDs in Bioimaging, Biosensing, Drug Delivery, Phototherapy, Antimicrobial Treatment, and Regenerative Medicine.

CDs can integrate multiple diagnostic and therapeutic functions into a single nanoscale platform, making them highly promising candidates for precision nanomedicine and image-guided therapy, as shown in Figure 5. In particular, CDs have shown remarkable potential in photodynamic therapy (PDT) and photothermal therapy (PTT). During PDT, photoactivated CDs generate ROS, including singlet oxygen and free radicals, which induce oxidative damage and apoptosis in cancer cells and microbial pathogens. In PTT, CDs efficiently convert near-infrared (NIR) or visible light into localized thermal energy, resulting in hyperthermia-mediated tumor ablation. The simultaneous combination of fluorescence imaging, ROS generation, photothermal conversion, and targeted therapeutic delivery within a single nanoplatform enables synergistic theranostic performance with enhanced treatment precision and monitoring capability. Consequently, the multifunctional biomedical behavior of CDs highlights their growing translational potential for advanced cancer therapy, biosensing, regenerative medicine, antimicrobial treatment, and personalized healthcare applications.

Compared with other carbon-based and inorganic nanomaterials, CDs occupy a distinct position in biomedical applications. As summarized in Table 3A, graphene oxide and carbon nanotubes provide high surface area, strong drug-loading capacity, and photothermal performance, but their larger size, slower clearance, and potential inflammatory effects may limit clinical translation. MOF-derived carbons and activated porous carbons offer excellent porosity and adsorption capacity but generally lack intrinsic fluorescence for real-time imaging. Metal oxide nanoparticles provide magnetic, catalytic, or photothermal properties but may raise concerns regarding metal ion release and long-term toxicity. In contrast, CDs combine intrinsic fluorescence, water dispersibility, low-cost synthesis, surface tunability, and potential renal clearance, making them especially useful when imaging, sensing, and therapy need to be integrated into one platform.

In biosensing, CDs act as fluorescent or electrochemical probes for detecting metal ions, glucose, reactive oxygen species, DNA, miRNA, proteins, and disease biomarkers. Their sensing performance is mainly governed by fluorescence quenching/enhancement mechanisms, including Förster resonance energy transfer, photoinduced electron transfer, inner filter effect, and surface-state modulation. For phototherapy, CDs can generate reactive oxygen species for PDT or localized heat for PTT under light irradiation. NIR-active CDs are particularly promising because they improve tissue penetration and allow fluorescence-guided treatment. In antimicrobial and wound-healing applications, CDs may contribute to these effects through membrane disruption, ROS generation, pH sensing, and scaffold reinforcement. These major biomedical applications, mechanisms, design requirements, and limitations are summarized in Table 3B.

In addition, the use of CQDs as functional components is also gaining momentum in other biomedical matrices, such as hydrogels, polymer networks, metal oxides, silica nanoparticles, MOFs, liposomes, electrospun fibers, and wound dressings. In these composite systems, the CQDs serve as more than just a filler: They are used for fluorescence tracking, drug loading, ROS modulation, pH sensing, antibacterial activity, electron transfer, or photothermal/photodynamic. For instance, CQD–polymer hydrogels can enhance the mechanical stability, injectability, pH-sensitive drug release, and real-time fluorescence monitoring. Fluorescence imaging, magnetic response, and/or catalytic activity, and/or tumor microenvironment-responsive therapy can be integrated into CQD–metal oxide composites, e.g., Fe_3_O_4_@CQD or MnO_2_/CD systems [141]. CQD–MOF composites have the potential to combine the advantages of the high porosity and drug-loading capacity of MOFs with the optical traceability and surface tunability of CQDs. Hence, CQD-based composites can be superior to pristine CQDs if the base material is superior in terms of loading capacity, stability, targeting, or multimodal therapeutic performance. But composites can also be more synthetic, less reproducible, slower to clear, more toxic, and pose greater regulatory issues, so the superior nature of composites must be considered based on the biomedical application [142].

Earlier studies reveal that many CD-based systems exhibit potent in vitro fluorescence, drug loading, biosensing, or cytotoxicity properties; however, fewer studies provide full translational evidence. However, in vitro fluorescence is not always correlated with in vivo imaging due to various factors, such as tissue autofluorescence, protein corona formation, immune recognition and renal/hepatic clearance, which can affect signal intensity and biodistribution. Likewise, if a drug-loading capacity is high, it must be accompanied by release kinetics, a lack of premature leakage, targeting specificity, a therapeutic index and safety under physiological conditions. Standardized physicochemical characterization, purification procedures, serum stability, hemocompatibility, immunotoxicity, pharmacokinetics, biodistribution, organ clearance, repeated-dose toxicity and comparison with clinically used nanocarriers should therefore be included in future studies. CDs are promising nanoplatforms for diagnostics and therapeutic use, but to be successfully translated into the clinic, reproducible synthesis, rational surface engineering, biological stability, and application-specific safety evaluation will be crucial [30].

### 5.1. Surface Modification, Targeted Delivery, and Biocompatibility–Toxicity Considerations

Surface modification is critical for enhancing the biomedical functionality of CDs, especially in targeted drug-delivery systems. Covalent or noncovalent functionalization can be used to introduce functional groups like amino, carboxyl, hydroxyl, sulfonate, and conjugate drugs, genes, or targeting ligands with specific receptors on the cancer cells to improve cellular uptake [143,144]. Such modifications can also facilitate stimulus-responsive drug delivery (e.g., pH-responsive delivery in an acidic tumor microenvironment), thereby improving therapeutic efficacy and reducing off-target toxicity [143,145]. Surface functionalization in gene therapy improves the stability and dispersibility of nanoparticles in biological fluids and enhances imaging and theranostic functions by combining diagnostics with therapy into a single platform through metal doping and ligand engineering [146,147].

Even though CQDs are regarded as biocompatible, their toxicity is highly influenced by the dose, surface chemistry, and exposure conditions. In a number of studies, low toxicity and high in vivo tolerance have been observed to date, and cytotoxicity and biochemical stability at animal models of imaging doses [125,148]. However, exemptions also exist: highly cationic or functionalized CQDs of this type may induce hepatotoxicity, inflammation, or cell death, and multiple doses of it can lead to systemic toxicity in animal models [149,150]. Long-term light exposure can reduce the quality of CDs to potentially poisonous products, putting cytotoxic pressure [151]. CDs can be used in clinical translation; their safety should be assessed at the formulation, dose, and application level, and long-term toxicity studies remain to be conducted before the widespread clinical adoption of these compounds [152,153].

### 5.2. Comparative Analysis of Doping Versus Surface Functionalization Effects on CQDs

Doping and surface functionalization are both important for improving CQDs for biomedical imaging; however, they influence CQDs in different ways. Heteroatomic doping with nitrogen, sulfur, phosphorus, or metals is the main way of altering the electronics of CQDs; this positively influences photoluminescence quantum yield, photostability, and allows new optical behaviors, such as upconversion luminescence and multimodal imaging performance, which improves brightness and imaging contrast when used in bioapplications [154,155]. Surface functionalization, in contrast, entails the modification of CQDs with polymers, targeting ligands, or other molecules to enhance biocompatibility, stability, and cell- or tissue-specific targeting, which is key to accurate imaging-guided drug delivery and tumor diagnostics [156,157,158]. Although doping is the primary method for controlling intrinsic optical characteristics by altering the core or edge states of CQDs, surface functionalization offers extrinsic control over biological interactions and targeting specificity [159,160]. In fact, co-doping and surface functionalization can yield synergies, with doped CQDs in the presence of specific surface ligands being more responsive in fluorescence, exhibiting enhanced cellular uptake and lower toxicity [125,161]. In total, doping and surface functionalization of CQDs are used to optimize the basic photophysical properties required for signal generation in medical imaging and to achieve targeted delivery and biological compatibility for biomedical imaging [19,162].

### 5.3. Doping and Surface Functionalization Effect on CQDs

Both doping and surface functionalization play important roles in the luminescence of CQDs, but in dissimilar ways. In a large proportion of cases, hetero-doping (e.g., with nitrogen) can alter the surface states and electronic structure of CQDs, and in most cases, enhance the photoluminescence quantum yield by creating new emissive sites, passivating defects, or by altering both core and surface states; in one study, a 33 percent increment of quantum yields can be obtained with nitrogen doping [67,163,164]. Conversely, surface functionalization has a major impact on fluorescence by passivating surface defects and functional groups that can modify photoluminescence surface traps but do not affect the core structure [165,166]. Research also shows that one of the primary sources of emission in doped CQDs is hybridized surface states rather than the core, and that surface chemistry has a dominant effect on fluorescence [163]. It is common to combine surface functionalization with doping to realize synergistic effects, which are brighter and more stable when intrinsic electronic properties are simultaneously tuned and when extrinsic surface chemistry is [167,168]. On the whole, the intrinsic optical properties are altered by doping, which alters the electron state, whereas fluorescence is regulated by surface functionalization through chemical passivation and environmental interactions [169,170].

### 5.4. Role of Sulfur and Nitrogen Codoping to Alter Carbon Dot Photoluminescence

ulfur and nitrogen (S, N) co-doping is a well-established and effective strategy for tuning carbon dot (CD) photoluminescence because these heteroatoms synergistically modify both the electronic structure of the carbon core and the nature of surface emissive states. Nitrogen introduces pyridinic, pyrrolic, graphitic, and amine-related states that enhance electron donation, defect passivation, and radiative recombination. Sulfur introduces C–S, C=S, thiol, and –SCN-related states that redistribute charge density and promote red-shifted or stabilized emission [70,108]. The combined effect typically yields higher photoluminescence quantum yield (PLQY), improved aqueous stability, and tunable excitation-dependent or excitation-independent behavior [171]. For example, S, N-CQDs synthesized hydrothermally from citric acid and thiosemicarbazide exhibited strong emission at 430 nm (λex = 360 nm) and served as sensitive fluorescent probes for pharmaceutical nitro compounds (rifampicin, metronidazole, ornidazole, tinidazole), with detection limits in the sub-micromolar to low micromolar range [172]. In another well-cited system, N, S-CQDs prepared from citric acid and thiourea achieved a PLQY of up to 53.80% and demonstrated selective, sensitive Fe^3+^ detection, illustrating how codoping can simultaneously optimize fluorescence efficiency and sensing performance [173]. The highest PLQY reported for this class 73% was achieved using citric acid and L-cysteine as precursors, attributed to the synergistic suppression of non-radiative pathways through simultaneous N and S surface passivation [34,136]. S, N-CQDs have further been explored for red-shifted emission: using citric acid and thiourea in dimethylformamide (solvothermal), red-emitting CDs with peak emission at 610 nm and PLQY of ~24% were obtained, with sp^2^-domain conjugation and surface nitrogen engineering identified as the primary determinants of the red emission [174]. Notably, however, S, N-codoping does not automatically guarantee red/NIR emission or clinical utility; the final optical behavior is critically determined by precursor ratio, reaction temperature, solvent polarity, surface oxidation state, and aggregation state [70,171]. Therefore, S, N-codoped CDs should be treated as tunable structure–property systems rather than a uniformly superior class, and their design must be guided by explicit mechanistic understanding of how each synthesis parameter modulates dopant bonding configuration and emission center distribution.

## 6. Evaluation of Toxicity, Biocompatibility, and Safety of Carbon Dots (CDs)

Although carbon dots are generally considered biocompatible and low in toxicity, their safety largely depends on the synthesis method, surface chemistry, particle size, dopants, concentration, and biological environment. Recent studies emphasize that CD toxicity is formulation-dependent and should not be generalized [175]. At the cellular level, toxicity may arise from oxidative stress, membrane damage, mitochondrial dysfunction, and inflammation, particularly in highly cationic or PEI-modified CDs. In contrast, PEGylated or neutrally charged CDs often show improved cytocompatibility. In vivo behavior is influenced by biodistribution, surface charge, colloidal stability, and clearance pathways, with larger or aggregated CDs potentially accumulating in organs such as the liver and spleen. Therefore, comprehensive toxicity evaluation, including cytotoxicity, ROS generation, biodistribution, and long-term safety studies is essential before clinical translation. Overall, CDs remain highly promising for biomedical applications, but their safety must be assessed on a case-by-case basis rather than assuming universal biocompatibility [175].

### 6.1. General Toxicity Profile and Biocompatibility of Carbon Dots

Carbon dots (CDs) are typically low-toxic and well-biocompatible, and therefore have potential applications in a wide range of biomedical applications, such as bioimaging, drug delivery, biosensing, and phototherapy. Their toxicity, however, is highly dependent on physicochemical characteristics such as surface functionalization, size, charge, and concentration. For example, cell morphology and the regular progression of the cell cycle remain unaffected in neutral polyethylene glycol (PEG)-modified CDs, which exhibit low cytotoxicity at relatively high concentrations. On the contrary, positively charged CDs, especially those with polyethylenimine (PEI) on their surface, exhibit greater cytotoxicity due to increased cellular uptake and interactions with intracellular components, often leading to cell cycle disruption [176]. The studies conducted both in vitro and in vivo indicate that CDs are generally safe at controlled doses. Nitrogen-doped, folic acid-modified functionalized CDs exhibit minimal biochemical, hematological, and histopathological changes in animal models [177]. Biomass-based CDs, which are fabricated from natural compounds, are also more biocompatible due to their less harmful synthesis pathways and lower concentrations of toxic byproducts [153,178]. CDs can be relatively water-soluble, chemically stable, and photobleachable, which are useful for biological applications.

**Figure 6:**
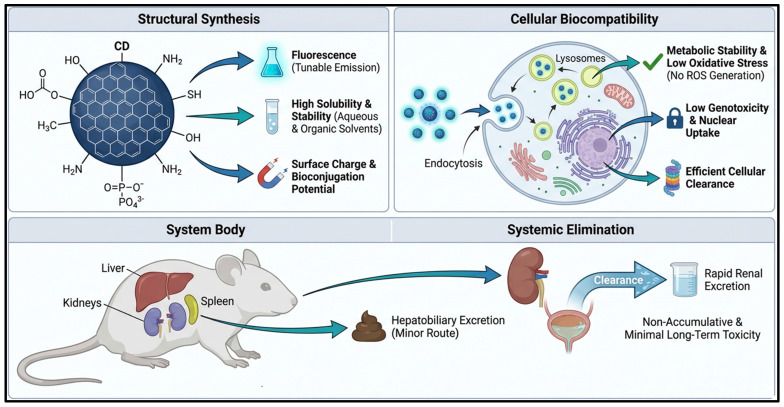
Structure–Property Relationship and Comprehensive Toxicity Profile of CDs: Insights into Biocompatibility, Cellular Response, and In Vivo Safety Mechanisms.

Toxicity and biocompatibility of CDs are governed by a well-defined structure-property relationship, in which surface chemistry, functional groups, and surface charge are critical determinants of biological outcomes. CDs have a variety of surface functionalities (–OH, –COOH, –NH2, and phosphate groups) that directly affect their fluorescence properties, aqueous solubility, and bioconjugation efficiency, as seen in Figure 6. The cumulative action of these physicochemical properties is the regulation of cellular interaction pathways and systemic fate. At the cellular level, CDs are primarily internalized via endocytosis and localized in lysosomes. PEG-functionalized and biomass-based CDs are more biocompatible, with low ROS generation, minimal oxidative stress, intact mitochondrial integrity, and no genotoxicity. Conversely, PEI-tagged CDs exhibit increased cellular uptake but can cause intracellular stress due to robust electrostatic interactions with cellular membranes, leading to increased ROS generation and partial mitochondrial dysfunction.

At the systemic level, studies on biodistribution show that CDs are primarily concentrated in reticuloendothelial organs, particularly the liver, spleen, and kidneys. Additionally, it is noted that renal excretion is the major route of clearance, with hepatobiliary excretion as a secondary route, thereby ensuring effective systemic elimination of most CD preparations. Well-engineered CDs typically accumulate slowly over a long period, which is why they have a positive in vivo safety profile. Although these are promising findings, some CDs have been known to cause mild oxidative stress or transient biochemical disturbances, depending on dose and surface chemistry. Thus, rational surface engineering is the most important factor in balancing biomedical functionality and safety. The optimization of synthesis strategies and surface modifications should be carefully carried out to ensure safe clinical translation [176,179,180].

### 6.2. Mechanisms of Toxicity and Cellular Interactions

**Figure 7:**
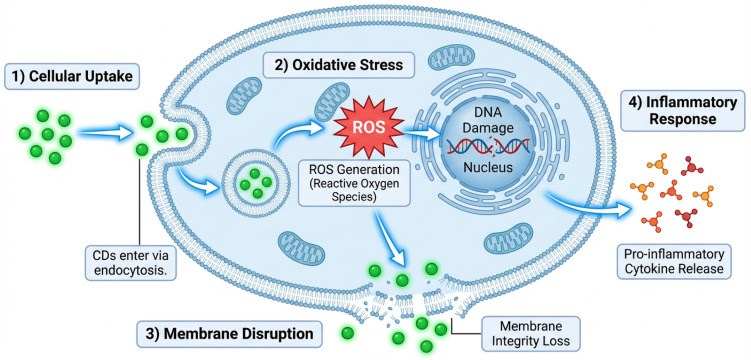
Toxicity and Cellular Interactions of CD Mechanisms.

CD toxicity primarily involves oxidative stress, membrane perturbation, and genetic damage, as shown in Figure 7. One of them is the formation of reactive oxygen species (ROS), which can lead to lipid peroxidation, protein denaturation, and DNA damage. This oxidative stress increases membrane permeability, leading to cellular swelling (oedema) and, subsequently, cell death, as shown in bacterial and mammalian models [181]. The other significant cause of toxicity is photo-degradation. On light exposure, CDs may break down into smaller reactive molecules and free radicals, which increase cytotoxicity, especially in human cells [182].

Surface charge has a profound effect on these interactions: positively charged CDs can enter the nuclear membrane and interact directly with DNA, thereby increasing cytotoxicity and altering cell cycle regulation. On the contrary, neutral and negatively charged CDs usually do not leave the cytoplasm, and they are not highly toxic, although, again, they can cause oxidative stress [176,183]. Zebrafish embryos exposed to high doses of CDs in vivo exhibit developmental defects, which are closely associated with oxidative stress and disruptions in lipid metabolomics [182,184]. Moreover, oxygen- and nitrogen-containing functional groups on the CD surface can increase ROS production and toxicity, and this effect may increase with repeated exposure [149]. These results indicate that the main processes underlying CD toxicity are ROS generation, membrane contact, nuclear penetration, and photochemical instability.

### 6.3. In Vitro and in Vivo Toxicological Studies and Surface Modification Effects

**Figure 8:**
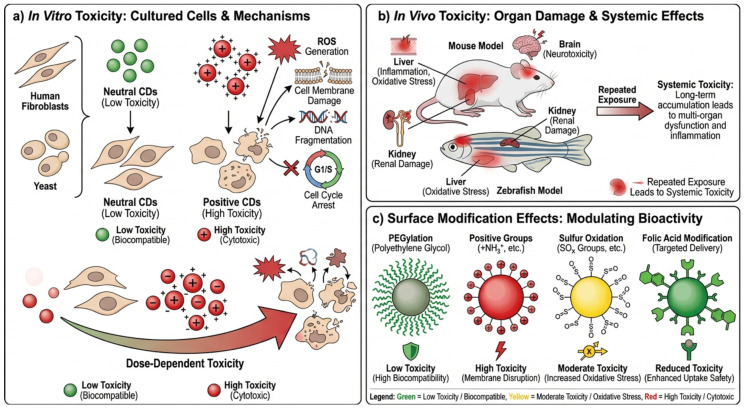
Toxicological Effects of CDs: In Vitro, In Vivo, and Surface Modification-Dependent Responses. (**a**) In Vitro Cytotoxicity and Cellular Responses Induced by CDs. (**b**) In Vivo Toxicity and Organ-Specific Effects of CDs in Animal Models. (**c**) Impact of Surface Functionalization on the Biocompatibility and Toxicity of CDs.

In vitro experiments consistently indicate that CDs are relatively non-cytotoxic to cells in the short term. However, different surfaces can become highly toxic depending on surface functionalization and charge, as shown in Figure 8a. The CDs with a neutral charge are generally the least toxic, positively charged are more toxic, entering the nucleus and causing oxidative stress and cell cycle arrest in fibroblasts and other cells [176,183]. The cytotoxicity data of various biological models, such as yeast and human cell lines, show that there are dose-dependent effects related to the production of ROS, the damage of membranes, and DNA fragmentation, which can be increased by longer exposure or light exposure [181,185].

In vivo experimentation provides greater insight into systemic toxicity, as detailed in Figure 8b. Mice experiments indicate that the repeated use of CDs with oxygen-based and nitrogen-based surface groups can cause lethality and organ-specific toxicities like renal tubule damage and liver blood flow impairment, even though survivors typically do not damage their organs severely [149]. Hepatotoxicity testing shows that positively charged CDs can cause liver inflammation and oxidative stress, and it is important to note that surface charge was a critical factor influencing biological results [186]. Likewise, zebrafish models exhibit developmental toxicity, neurotoxicity, and organ damage at high concentrations of CD [187].

Surface modification is decisive in the modulation of the CD safety. PEGylation has a profoundly positive effect on biocompatibility, decreasing the nonspecific interaction and uptake by cells, whereas positively charged functionalization (PEGs, PEI, spermidine) raises cytotoxicity and inflammatory reactions [188,189]. Oxidation of sulphur functional groups to cysteamine-functionalized CDs increases oxidative stress and in vivo toxicity further than unmodified versions or folic acid-modified CDs [190] as detailed shown in Figure 8c. CDs are widely considered safe nanomaterials; their toxicity is highly context-dependent. Key determinants include surface chemistry, charge, concentration, exposure duration, and dosing frequency. The issue of long-term toxicity and bioaccumulation is not adequately studied, which is why the need to implement standardized toxicity tests and design control strategies is urgent to ensure their safe use in clinical and environmental settings [191,192].

## 7. Challenges and Limitations

**Figure 9:**
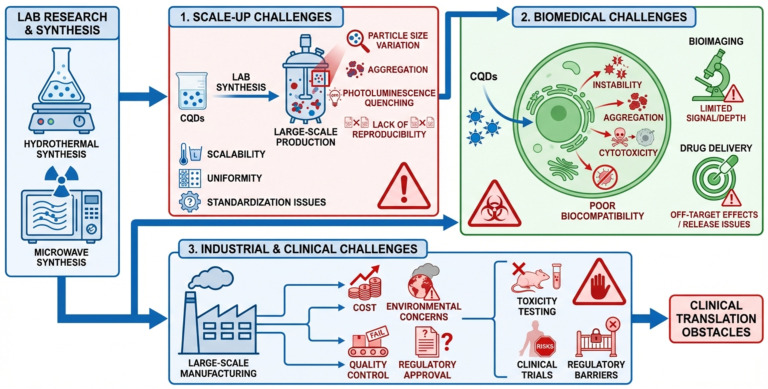
Challenges and Limitations in the Scale-Up and Clinical Translation of CQDs.

Key challenges limiting CQD translation include scaling production and maintaining quality, which prevent its translation into the commercial and clinical spheres [193,194] as detailed seen in Figure 9. The lack of standardization of synthesis techniques and characterization procedures leads to variability in CQD characteristics, making reproducibility and comparison across studies difficult [195,196]. Their long-term stability in applications such as bioimaging and sensing across various environmental conditions (e.g., photostability and chemical degradation) remains problematic and needs improvement [197]. The regulatory hurdles stem from limited knowledge of CQD’s toxicity, biocompatibility, and environmental impact, making it difficult to approve biomedical and environmental applications [198,199]. Also, there is a problem with optimizing surface functionalization to balance efficacy and safety, and to reduce production costs to make it widespread [200,201]. To solve these problems, improvements in scalable green synthesis, standardized protocols, more robust stability indicators, and a comprehensive safety analysis are needed to facilitate reliable CQD applications.

### Scale-Up, Manufacturing, and Clinical Translation Challenges of CQDs

One of the most important and least resolved challenges in the field is the ability to move CQDs from the lab to industrial and clinical production. Techniques like hydrothermal, microwave-assisted, and solid-state synthesis have been used to produce grams to kilograms of materials, but the goal of obtaining a particle size distribution, surface chemistry, and optical properties that are consistent on a batch-to-batch basis is still a basic challenge to overcome [22,202]. Variability in reaction conditions (e.g., temperature, precursor concentrations, mixing) during scaling-up will directly impact CQD size, quantum yield, and surface functional group density [22,196]. One promising solution is the continuous-flow reactor technology with a stationary reaction zone, which enables reproducible production rates up to 6.6 mg min^−1^, a narrow size distribution, and good optical properties [203]. Precise thermal and concentration-controlled microfluidic systems and optimized microwave platforms are also helpful in enhancing batch-to-batch reproducibility [202].

Another major challenge for bulk synthesis is aggregation-induced quenching of PL, which limits the optical properties of CQDs and makes them less attractive for biomedical applications. This self-quenching occurs when they are synthesized at higher concentrations [204,205]. Spatial confinement with metal cations, surface passivation with PEG or other hydrophilic polymers, and deliberate surface group modification to manipulate aggregation-induced emission (AIE) behavior are strategies that can be used to overcome this limitation [204,205]. Colloidal and optical stability of CQDs in biological systems, where the CQD is subjected to changes in ion concentrations, pH, and enzyme activity, is another layer of complexity that has not been comprehensively studied in laboratory settings [19,197].

Functionalizing the surface at a large scale is a different challenge. Precise chemical control that does not affect the fluorescence, colloidal, or biocompatibility properties of the molecules is required to introduce targeting ligands, therapeutic molecules, or protective coatings into the products, but it becomes more difficult as production volume increases [31,197]. Reproducibility of functionalization chemistry is especially important for biomedical applications because the properties of the chemistry directly impact the biodistribution, cellular uptake, and therapeutic specificity [206].

The most important remaining hurdles to clinical translation are safety and regulatory readiness. In general, the degree of cytotoxicity of CQDs is low at the tested concentration, but concerns are still raised on the toxicity of the photodegraded CQDs, dose-dependent effects, long-term biodistribution, and immunogenicity, which need to be carefully studied in vitro and in vivo prior to regulatory submission [182,207]. The regulatory framework for nanomaterials remains complicated and varies across regions, ranging from the U.S. Food and Drug Administration (FDA)/European Medicines Agency (EMA). There are no universally adopted characterization or quality control standards for products containing CQD. Standardized synthesis procedures, established characterization criteria, and reproducible toxicology assays are therefore urgently required to move laboratory innovations towards the clinical use of CQDs as safe and effective nanomedicine platforms [28,196].

## 8. Future Clinical Perspectives

Carbon quantum dots (CQDs) are a new class of nanomaterials with great potential for clinical translation in biomedicine. Owing to their unique physicochemical properties, such as high aqueous solubility, low cytotoxicity, excellent biocompatibility, photo stability, and tunable photoluminescence, they are highly applicable in advanced biomedical applications. Compared with conventional semiconductor quantum dots, CQDs are less toxic to the human body and safer for in vivo use. These features facilitate its integration into various clinical platforms, particularly for targeted drug delivery, real-time bioimaging, biosensing, and image-guided therapy.

Another advantage of CQDs is that their surface chemistry can be high-precision functionalized, and biomolecules such as peptides, antibodies, aptamers, and small-molecule ligands can be attached. This functional plasticity can be used to direct receptors to accumulate in diseased tissue, specifically tumors. CQDs have the potential to be incorporated into stimuli-responsive nanocarriers that can sense endogenous biological cues such as pH, redox, enzymatic, or temperature changes. This is site-specific drug delivery, which allows for controlled release and therefore a better response to treatment with less off-target toxicity.

CQDs can be used to design theranostic systems that combine therapeutic and diagnostic functions on a single nanoplatform, providing real-time therapeutic and diagnostic capabilities. They are naturally fluorescent and can be used in imaging large areas, while their functional surfaces can be loaded with drugs and delivered. This twofold capability enables real-time monitoring of therapeutic response, drug distribution and cellular uptake, which is important for precision medicine. CQD-based systems have proven effective for tumor imaging contrast, increasing the efficacy of chemotherapy and photodynamic and photothermal therapies in oncology.

CD development using AI should not be marketed as a proven clinical solution, but as a developing optimization tool. Machine learning can be used to uncover correlations between precursor type, precursor ratio, solvent, reaction temperature, reaction time, pH, dopant chemistry, and output properties of the particles, including emission wavelength, quantum yield, particle size, and sensing response. For instance, random forest, gradient boosting, support vector regression, polynomial regression, and neural networks have been applied in recent ML studies to predict CD photoluminescence and optimize synthesis conditions. The reported input parameters are the identity of the precursor, solvent, reaction time and reaction temperature, and the output parameters are the maximum emission wavelength, Stokes shift, and quantum yield.

The application of AI in CD nanomedicine is hindered, however, by limited dataset size, inconsistent reporting standards, batch heterogeneity, black-box model interpretation, and poor alignment between in vitro predictions and in vivo pharmacokinetics. In addition, recent ML studies highlight the importance of improving data set quality to enable better model development and increase model usefulness. Standardization of CD databases, validation of biological data, explainable models and experimental verification of AI-predicted designs will be needed for future clinical translation.

But this interdisciplinary progress of nanotechnology, computational science, and biomedical engineering is slowly addressing these challenges. The future of precision oncology, advanced diagnostics, and personalized therapeutics is expected to be largely defined by the CQDs, particularly with the incorporation of AI into system design and optimization. They have taken a significant step towards next-generation, intelligent healthcare systems by developing experimental nanomaterials into clinically viable systems.

## 9. Conclusions

CDs have emerged as one of the most scientifically compelling and structurally versatile nanomaterial platforms in contemporary nanomedicine, offering a uniquely favorable combination of sub-10 nm particle dimensions, tunable photoluminescence across the visible-to-near-infrared spectrum, rich surface chemistry amenable to precise bioconjugation, and an inherently low toxicity profile that collectively distinguishes them from conventional heavy-metal-based semiconductor quantum dots. Their rational design for clinical applications demands stringent physicochemical and biological property sets, to which carbon-based nanoarchitectures are increasingly being tailored for targeted drug delivery, real-time bioimaging, biosensing, photodynamic and photothermal therapy, and theranostic platforms. As earlier studies show, a few issues remain to be addressed for true clinical translation. The origin of the CD photoluminescence, related to either quantum confinement, surface defect states, molecular fluorophore centers, or crosslinked polymer cores, is incompletely understood in many reported systems, directly impeding rational optimization of quantum yield, emission wavelength, and photostability. Deliberate heteroatom doping strategies, precise surface passivation, and thorough mechanistic verification are necessary to achieve consistent red and near-infrared emission with high quantum efficiencies in physiological aqueous media, as well as to enable stimuli-responsive drug release, active receptor-mediated targeting, and well-defined in vivo pharmacokinetics, rather than trial-and-error synthesis. There are several translation barriers that need to be addressed systematically through quality-by-design synthesis principles, standardized characterization protocols, and clinically relevant biological models, such as batch-to-batch synthetic heterogeneity, product purity standards, limited scalable manufacturing, incomplete long-term toxicological data, and the lack of CD-specific regulatory frameworks. The careful addition of ML- and AI-driven optimization tools to CD design workflows presents a complementary approach that, if grounded in carefully curated, high-quality experimental data and backed by rigorous experimental validation, can prove viable. Instead, CDs (and any other nanomaterials) should be viewed as platforms with complex structures that can be tuned by the precision of their fabrication, the mechanistic depth of their characterization, and the biological rigor of their evaluation, and whose clinical promise is directly proportional to these. If these requirements are addressed, CDs may offer substantial promise in the coming decades for precision oncology, advanced diagnostics, and personalized medicine.

## Figures and Tables

**Table 1:** Representative CD synthesis routes: mechanistic features, key parameters, and optical/biological outcomes.

Synthesis Route	Representative Example	Precursors/Conditions	Core Mechanistic Step	QY/Optical Outcome	Key Advantage	Key Limitation	Citations
Hydrothermal (N-doping)	Citric acid + urea or ethylenediamine, 160–200 °C, 4–12 h	Citric acid (C source); urea/EDA (N source); water solvent	Carbonization + N-passivation introduces pyridinic/pyrrolic surface states; suppresses non-radiative decay	QY: 26.6–35.08%; blue emission (~440–445 nm); excitation-independent	High PL stability; strong water dispersibility; simple one-pot route	Long reaction time; batch-to-batch variation with temperature fluctuation	[68,69]
Hydrothermal (S, N codoping)	Citric acid + thiourea or cysteine, 160–200 °C	Citric acid (C source); thiourea/cysteine (N and S source)	N/S synergy creates new emissive surface states; C–S and C=S bonds modulate charge distribution and radiative recombination	QY: up to 73% (cysteine); blue-green emission; reduced excitation dependence	Superior QY; improved charge transfer; negligible cytotoxicity	Sulfur source choice critically affects emission color and QY; complex surface chemistry	[70]
Green/Biomass synthesis	Garlic, milk, rice straw, onion waste; hydrothermal or pyrolysis	Heterogeneous natural precursors containing C, N, O, S	Thermal decomposition of complex biomolecules; self-passivation from inherent heteroatoms	Variable QY (typically 5–20%); tunable emission depending on precursor	Sustainable, low-cost, reduced toxicity, renewable carbon sources	Precursor heterogeneity causes poor batch reproducibility, inconsistent optical properties, and difficult scale-up	[71,72]
Electrochemical oxidation	Graphite rod/carbon fiber electrode oxidation in aqueous electrolyte	Graphite/carbon electrode; NaOH, NaH_2_PO_4_, or H_2_SO_4_ electrolyte	Top-down electrochemical exfoliation generates surface-oxidized CDs rich in –OH, –COOH, and C=O groups	QY: typically, low to moderate (2–15 nm particle size); broad emission	Room-temperature synthesis; no organic solvents; controllable via applied potential	Broad size distribution; low QY without further passivation; limited scalability	[73,74]
Microwave-assisted synthesis	Citric acid + o-phenylenediamine or amino precursors; 3–10 min, household/lab microwave	Citric acid (C source); amine precursor; water or ethylene glycol	Rapid, uniform dielectric heating accelerates carbonization and surface functionalization	QY: 29–94.4%; blue emission; ~75% reduction in reaction time vs. hydrothermal	Fast, scalable, higher particle yield in a shorter time	Overheating increases inter-layer spacing heterogeneity; it is difficult to control uniformly; there is a risk of incomplete carbonization	[75,76]

**Table 2:** Integrated Analysis of CDs from Natural and Synthetic Sources Including Characterization Techniques, Key Physicochemical Findings, and Biomedical Applications.

Precursor/Synthesis	Size/Morphology	Key Characterization Findings	Techniques Used	Optical Properties	Surface Chemistry	Structure (XRD)	Biomedical/Medical Relevance	Citations
Epigynum auritum (hydrothermal)	3–8 nm, spherical	Strong NIR emission, high tissue penetration, low toxicity	UV–Vis, Fluorescence, TEM, XPS	NIR fluorescence	–OH, –COOH	Amorphous	Deep-tissue bioimaging, antimicrobial (ROS generation)	[86]
Folic acid CDs	2–6 nm	Tunable photoluminescence, high photostability	UV–Vis, FL, FTIR, XRD	Blue–green emission	–NH_2_, –COOH	Graphitic domains	Cancer targeting via folate receptors, imaging	[87]
Functionalized CDs	~5 nm	Multicolor emission controlled by surface states	Fluorescence, TEM, FTIR	Excitation-dependent emission	Functional groups	Semi-crystalline	Cellular imaging, biosensing	[88]
Review	2–10 nm	Surface defects dominate fluorescence	FTIR, XPS, UV–Vis	Tunable emission	Variable groups	Amorphous	Drug delivery, sensing	[77]
Green CDs	3–7 nm	Eco-friendly synthesis, stable fluorescence	UV–Vis, FL, XRD	Stable emission	Oxygen-rich	Amorphous	Biocompatible nanomedicine	[89]
Resveratrol CDs	~4 nm	Improved solubility, enhanced bioactivity	FTIR, TEM, FL	Strong emission	Phenolic groups	Amorphous	Wound healing (enhanced bioavailability)	[90]
Curcumin CDs	2–5 nm	Antioxidant activity + multicolor fluorescence	UV–Vis, FL, TEM	Multicolor emission	Phenolic groups	Amorphous	Cancer detection, ROS scavenging	[91]
Review	2–10 nm	High quantum yield, photostability	UV–Vis, FL	Strong fluorescence	Functionalized surface	Mixed	Anticancer imaging	[92]
N, P, S-doped CDs	3–6 nm	High selectivity for metal ions	XPS, FL, TEM	Strong fluorescence	Heteroatom doping	Graphitic	Cancer imaging, biosensing	[93]
Coal CDs	5–10 nm	High structural stability	XRD, TEM, UV–Vis	Moderate fluorescence	Carbon-rich	Graphitic/amorphous	Drug detection, biosensing	[94]
Citric acid + cysteine	2–4 nm	High QY (>50%) fluorescence	UV–Vis, FL	Bright emission	–SH, –NH_2_	Amorphous	Dopamine sensing, imaging	[95]
Molasses CDs	~6 nm	Low-cost synthesis, stable fluorescence	UV–Vis, FL	Blue emission	–OH groups	Amorphous	Bioimaging	[96]
Electrochemical CDs	3–5 nm	Uniform size, stable optical properties	TEM, XPS, UV–Vis	Stable fluorescence	Oxygen groups	Graphitic	Cell imaging, Fe^3+^ sensing	[97]
Cumin CDs	4–7 nm	High biocompatibility, stable emission	FTIR, FL, TEM	Strong fluorescence	–COOH, –OH	Amorphous	Drug delivery, imaging	[98]
Aloe vera CDs	~5 nm	pH and temperature sensitivity	UV–Vis, FL, FTIR	Responsive emission	Oxygen groups	Amorphous	Biosensing, diagnostics	[99]
N, F-doped CDs	~3 nm	Multiphoton absorption, deep-red emission	UV–Vis-NIR, FL, TEM	Deep-red/NIR emission	F, N doping	Graphitic	Deep-tissue imaging	[100]
Urea CDs	4–8 nm	Dual photoluminescence behavior	FL, XRD	Dual emission	N-rich surface	Amorphous	Imaging probes	[101]
Review	2–10 nm	High solubility, low toxicity	Multi-techniques	Strong fluorescence	Hydrophilic groups	Mixed	Drug delivery, cancer therapy	[102]
Review	—	Optical stability and fluorescence	UV–Vis, FL	Stable emission	Functionalized	—	Theranostics	[103]
Review	—	Surface tunability critical	FTIR, XPS	Tunable emission	Engineered surfaces	—	Imaging, sensing	[104]
PDA-coated CDs	~10 nm	Photothermal conversion ability	TEM, XPS, UV–Vis	Combined optical + thermal	PDA coating	—	Cancer photothermal therapy	[105]
Fe-doped CDs	3–6 nm	pH-sensitive fluorescence	FL, XPS	High QY emission	Fe doping	Graphitic	Gastric imaging	[106]
CDs	2–6 nm	Very high QY (~80%)	UV–Vis, FL	Strong fluorescence	Passivated surface	Amorphous	Diagnostics, imaging	[107]
S, N-doped CDs	~5 nm	Red-shifted emission	FL, TEM	Red emission	S, N groups	Graphitic	Tumor imaging	[108]
Review	—	High photostability	Multi-techniques	Stable fluorescence	Functional groups	—	In vivo theranostics	[109]
Lycii Fructus CDs	~4 nm	Green synthesis, stable emission	UV–Vis, FL, TEM	Multicolor	Oxygen groups	Amorphous	Cell imaging	[110]
Onion waste CDs	3–6 nm	Eco-friendly, high stability	UV–Vis, FL	Strong fluorescence	–COOH groups	Amorphous	Biosensing, imaging	[111]
N, S CDs	~5 nm	pH-sensitive fluorescence	FL, XPS	QY ~35%	Functional groups	Amorphous	Cell imaging, sensors	[112]
N, S CDs	~4 nm	Low toxicity, stable emission	FL, TEM	Strong fluorescence	Doped surface	Amorphous	Cancer imaging	[113]
Review	—	High fluorescence + stability	UV–Vis, FL	Tunable	Functional groups	—	Drug delivery, imaging	[114]

**Table 3:** (**A**) Comparative performance of carbon-based and related nanomaterials for biomedical applications. (**B**) Biomedical Applications of CDs/CQDs: Functional Mechanisms, Engineering Strategies, Biological Models, and Translational Challenges.

(**A**)									
**Material**	**Main Biomedical Strength**		**Typical Biomedical Use**	**Key Advantages**		**Limitations Compared with CDs**			**Citations**
CDs/CQDs)0-D, <10 nm	Intrinsic fluorescence, ultrasmall size, water dispersibility, tunable surface chemistry		Bioimaging, biosensing, drug/gene delivery, PDT, PTT, organelle tracking	Low cytotoxicity, Easy functionalization, Optical tracking, Biocompatible, Scalable green synthesis		Low red/NIR QY in many systems. Batch-to-batch variability: Emission mechanism debated			[19,115,116]
Graphene quantum dots (GQDs)0-D, graphene-derived	Graphene-like lattice, pronounced edge-state emission, high π-conjugation		Fluorescence imaging, electrochemical sensing, phototherapy and drug delivery	High surface area, Strong π–π stacking, High conductivity		Possible oxidative stress/ROS Synthesis complexity, less tunable optical emission than CQDs			[115,117]
Graphene oxide (GO)2-D nanosheet	Large 2-D surface with abundant drug-loading sites via π–π stacking and covalent bonding		Drug/gene delivery, photothermal therapy, antibacterial coatings, tissue scaffolds	High drug loading, pH-responsive release, NIR photothermal conversion		Larger size limits cell penetration. Long-term retention/toxicity concerns: Cytotoxic at high concentrations			[21,118,119]
Carbon nanotubes (CNTs), 1-D, single-walled carbon nanotubes (SWCNTs)/multi-walled carbon nanotubes (MWCNTs)	High aspect ratio, strong NIR absorption, efficient cellular penetration, gene vector potential		Photothermal therapy (PTT), drug/gene delivery, biosensors, tissue engineering scaffolds	Strong NIR absorbance, high drug capacity (lumen filling), Raman-trackable in vivo		Biopersistence and inflammatory concerns. Biotoxicity/immunotoxicity: Difficult to purify and disperse			[21,118,120]
Activated/porous carbon3-D, high-surface structure	Extremely high surface area and adsorption capacity; effective toxin removal platforms		Haemoperfusion, toxin removal, drug adsorption and adsorbent biosensor support	Excellent adsorption capacity, low-cost, scalable production		Weak intrinsic fluorescence, Poor imaging ability, Broad size distribution			[118,121]
MOF-derived carbons/CD@MOF hybrids, Porous nano-composite	Extraordinary porosity, tunable pore chemistry, strong drug encapsulation and catalytic activity		Drug loading & controlled release, biosensing, photocatalysis, cancer theranostics	High pore volume Structural tunability Optical + adsorptive synergy (CD@MOF)		Less intrinsic bioimaging than pure CDs. Stability in physiological media. Complex synthesis; scale-up challenging			[122,123]
Metal oxide nanoparticles (e.g., Fe_3_O_4_, TiO_2_, ZnO)0-D inorganic	Magnetic, catalytic, and photothermal properties enabling multimodal therapy and imaging		MRI contrast, magnetic hyperthermia, ROS/photodynamic therapy, targeted drug delivery	Strong magnetic response (Fe_3_O_4_), High ROS generation, Well-established MRI contrast		Metal ion leaching/toxicity, Potential systemic accumulation, Lower biocompatibility than CDs			[21,115]
Polymer/biomaterial nanoparticles poly(lactic-co-glycolic acid) (PLGA), chitosan)Organic polymer matrix	Biodegradability, clinical translation familiarity, sustained and stimuli-responsive drug release		Drug delivery, tissue engineering, gene therapy, vaccine adjuvants	Good biocompatibility, clinically established (PLGA), Tunable release kinetics		Require external fluorescent labels, Limited intrinsic optical functionality, Batch stability challenges			[116]
(**B**)									
**Application Domain**	**Specific Application/Target**	**Key Mechanisms/Functional Principles**	**Design/Engineering Requirements**	**Biological Models/Systems**	**Major Advantages**		**Limitations/Challenges**	**Representative Findings/Notes**	**Citations**
Bioimaging (In Vitro)	Cellular & organelle imaging (HeLa, MCF-7, A549, HepG2)	Excitation-dependent photoluminescence (PL); surface-state emission; endocytosis uptake; organelle targeting	Size <10 nm; surface passivation; functional groups; targeting ligands; multicolor/NIR tuning	Cancer cell lines; primary cells	High photostability; low cytotoxicity; multiplex imaging; real-time visualization		Low quantum yield in red/NIR; batch variability	Bright intracellular fluorescence with minimal photobleaching	[102,124,125]
Bioimaging (In Vivo)	Whole-body imaging; tumor tracking; organ imaging	Enhanced permeability & retention (EPR); first and second near-infrared windows (NIR-I/NIR-II) fluorescence; renal clearance	Ultra-small size (<5–8 nm); hydrophilic coatings; stable emission	Mouse, zebrafish models	Deep tissue penetration; rapid clearance; low background signal		Limited NIR-II efficiency; incomplete biodistribution data	Tumor localization and organ imaging with low toxicity	[109,126]
Advanced/Multimodal Imaging	FL/MRI/computed tomography (CT)/photoacoustic (PA) imaging	π-conjugation extension; heteroatom/metal doping (Gd, Mn, Fe, Bi)	Controlled doping; colloidal stability; toxicity shielding	Animal tumor models	Multimodal diagnostics; enhanced contrast		Synthetic complexity; metal toxicity concerns	Dual-mode fluorescence–MRI/CT tumor imaging	[127,128]
Optical Biosensing	Glucose, ROS, metal ions	PL quenching/enhancement (FRET, photoinduced electron transfer (PET), inner filter effect (IFE); surface recognition	Functionalized surfaces; ratiometric probes	Blood, serum, urine	High sensitivity (µM–nM); rapid response		Matrix interference; reproducibility issues	Detection of glucose, H_2_O_2_, Fe^3+^ in biofluids	[129,130]
Biomolecular Sensing	DNA, miRNA, proteins (PSA, thrombin)	Aptamer/antibody binding; hybridization-induced PL change	Stable bioconjugation; selective probes	Clinical samples; cell lysates	High specificity; label-free detection		Interference in complex matrices	Oncogenic DNA and protein biomarker detection	[102,124]
Electrochemical Biosensing	Glucose, biomarkers	Electron transfer enhancement; nanozyme activity	Doped CDs; conductive substrates	Wearable devices; fluids	Low detection limits; portable sensing		Fouling; long-term stability	Integration into wearable biosensors	[126,131]
Drug Delivery (Small Molecules)	Chemotherapy (e.g., doxorubicin)	π–π stacking; electrostatic/covalent loading; pH/redox release	Size 5–20 nm; targeting ligands; controlled loading	Tumor-bearing mice; cell lines	Controlled release; improved solubility		Dose standardization issues	Enhanced tumor inhibition vs. free drug	[28,132]
Targeted Drug Delivery	Tumor-specific delivery	Ligand-receptor targeting (folate, RGD, antibodies)	Surface functionalization; receptor specificity	Cancer models	Reduced off-target toxicity		Ligand instability in vivo	Folate-CDs accumulate in tumors	[29]
Stimuli-Responsive Delivery	Controlled drug release	pH, redox, enzyme-triggered release; phototriggering	Smart linkers; tumor microenvironment sensitivity	Tumor models	On-demand release		Premature leakage risk	Acidic pH-triggered release	[116,132]
Gene Delivery	small interfering RNA (siRNA), microRNA (miRNA), DNA	Electrostatic complexation; endosomal escape	Cationic coatings (PEI/chitosan); low toxicity	Cancer, neuronal models	Non-viral; imaging capability		Lower efficiency vs. viral vectors	Effective gene silencing	[133]
Vaccine/Antiviral Delivery	Immunotherapy platforms	Antigen conjugation; immune activation	Stable antigen binding; immune tuning	Preclinical immune models	Enhanced immunogenicity		Limited clinical translation	CDs used in antiviral vaccines	[116,134]
Photodynamic Therapy (PDT)	Cancer & antibacterial PDT	ROS generation (^1^O_2_, •OH, O_2_•−)	High ROS yield; light absorption; targeting	Tumor & infection models	Spatially controlled therapy		Limited light penetration	Tumor and bacterial ablation	[19,125]
Organelle-Targeted PDT	Mitochondria/nucleus targeting	Targeted ROS generation	Organelle-specific ligands	Cancer cells	High efficiency apoptosis		Targeting precision issues	Effective at low light doses	[29]
Photothermal Therapy (PTT)	Tumor ablation	NIR absorption; heat generation	High photothermal conversion; NIR tuning	Tumor models	Rapid, non-invasive therapy		Overheating risk	Tumor regression at >60 °C	[133]
Combination Therapy (Theranostics)	PDT + PTT + chemo	Synergistic ROS + heat + drug action	Multifunctional design; NIR response	In vivo tumor models	Enhanced efficacy; reduced recurrence		Complex synthesis	Significant tumor inhibition	[135]
Imaging-Guided Therapy	Theranostic systems	Integrated imaging + therapy	Multimodal platforms; targeting ligands	Animal models	Real-time monitoring		Regulatory challenges	Single platform diagnosis & therapy	[109,128]
Antibacterial Applications	Gram+/Gram− bacteria	ROS generation; membrane disruption; PTT/PDT	Surface charge tuning; light activation	Infection models	Broad-spectrum; low resistance		Limited in vivo data	Effective bacterial killing	[83,103]
Antiviral Applications	Viral inhibition	Entry/replication inhibition; ROS	Functionalized targeting	Viral models	Localized antiviral action		Limited mechanistic clarity	Potential in respiratory viruses	[116]
Tissue Engineering	Scaffolds & hydrogels	Mechanical reinforcement; ROS modulation; fluorescence tracking	Polymer integration; bioactive surfaces	Fibroblast, osteoblast models	Enhanced cell growth		Unknown long-term degradation	Supports tissue regeneration	[136]
Bone Regeneration	Osteogenesis	Differentiation promotion; nanozyme activity	Ca^2+^ binding groups; surface tuning	Bone models	Bone repair + imaging		Mechanism unclear	Increased mineralization	[137]
Wound Healing	Smart dressings	ROS modulation; pH sensing	Hydrogel integration; antimicrobial design	Skin models	Real-time monitoring; antibacterial		Stability issues	Multifunctional dressings	[138]
Brain/blood–brain barrier (BBB) Delivery	central nervous system (CNS) imaging & therapy	BBB penetration via size & surface	Ultra-small size; ligand engineering	Brain models	Brain targeting		Safety concerns	Effective BBB crossing	[19,138]
Green/Biomass CDs	Sustainable biomedical use	Biomass-derived synthesis	Green chemistry; reproducibility control	Zebrafish, mice	Eco-friendly; low cost		Batch variability	Effective imaging & therapy	[139]
General Platform Properties	Nanomedicine platform	Tunable PL (UV–NIR-II); high surface functionality	Surface engineering; doping; passivation	Broad biomedical systems	Versatile diagnostics & therapy		Lack of standardization	Widely applicable nanoplatform	[140]

## Data Availability

No new data were created for this manuscript.
